# Impact of a standardized oral care protocol on oral mucositis severity in leukemia patients receiving induction chemotherapy

**DOI:** 10.1097/MD.0000000000050630

**Published:** 2026-09-18

**Authors:** Fen Yu, Tian Mao

**Affiliations:** a Department of Hematology, Huanggang Central Hospital, Huanggang, Hubei, China; b Department of Nephrology, Huanggang Central Hospital, Huanggang, Hubei, China.

**Keywords:** CTCAE, induction chemotherapy, leukemia, opioid use, oral mucositis, pain, parenteral nutrition, retrospective cohort, standardized oral care protocol

## Abstract

This study aimed to evaluate whether a standardized oral care protocol is associated with reduced oral mucositis (OM) severity among leukemia patients receiving induction chemotherapy. Baseline characteristics were comparable between groups. Severe OM occurred in 17/90 (18.9%) in the protocol group versus 30/90 (33.3%) with usual care (odds ratio 0.46; *P* = .027); the association remained significant after adjustment (adjusted odds ratio 0.50; *P* = .044). OM duration was shorter with the protocol (median 6 vs 8 days; *P* = .003), with faster time to resolution (14 vs 16 days; *P* = .04). The protocol group reported lower maximum pain (median 3 vs 5; *P* < .001), less opioid use (44.4% vs 60.0%; *P* = .036), lower morphine milligram equivalents (60 vs 120 mg; *P* = .006), and reduced parenteral nutrition use (13.3% vs 24.4%; *P* = .049). Febrile neutropenia, blood culture positivity, intensive care unit transfer, and length of stay did not differ significantly. A standardized oral care protocol was associated with reduced severe OM and meaningful improvements in OM duration and symptom burden during leukemia induction chemotherapy. Prospective studies are warranted to confirm effectiveness and implementation fidelity. We conducted a single-center retrospective cohort study including adult leukemia inpatients receiving induction chemotherapy. Patients were grouped by exposure to a standardized oral care protocol with per-shift documentation versus usual care (90 vs 90). OM was graded each shift using the National Cancer Institute Common Terminology Criteria for Adverse Events (CTCAE) v5.0 from induction day 1 to hospital discharge. The primary outcome was peak OM severity; severe OM was defined as CTCAE grade ≥ 3. Secondary outcomes included OM incidence (CTCAE ≥ 1), time to onset, duration, time to resolution, maximum oral pain score (0–10 Numeric Rating Scale), opioid use and cumulative morphine milligram equivalents, parenteral nutrition use, febrile neutropenia (temperature > 38.3°C with neutropenia), blood culture positivity, intensive care unit transfer, and length of stay. Group comparisons used Pearson *χ*^2^/Fisher exact tests and appropriate parametric/nonparametric tests. Multivariable logistic regression assessed the association between protocol exposure and severe OM.

## 1. Introduction

Leukemia induction chemotherapy remains essential for achieving complete remission, but it is also characterized by intensive cytotoxic exposure and profound, sustained myelosuppression. In this setting, oral mucositis (OM) is one of the most frequent and clinically consequential toxicities. OM typically evolves from mucosal erythema to ulceration with severe pain, dysphagia, and impaired oral intake, which can compromise sleep, nutrition, and functional status. Contemporary supportive-care guidelines consistently highlight OM as a toxicity that not only worsens patient-reported outcomes but also increases the need for analgesics (including opioids), nutritional support, and inpatient resources, and may contribute to interruptions or modifications of anticancer therapy.^[1]^

The biological basis of OM supports its particular relevance in leukemia induction. Modern models conceptualize OM as a complex, multistage process involving epithelial injury, amplification of inflammatory signaling, and breakdown of mucosal barrier integrity, rather than a purely superficial local lesion.^[2]^ This cascade can be intensified by concomitant neutropenia and thrombocytopenia, which impair mucosal repair and increase bleeding and infection risk. In patients with hematologic malignancies, ulcerative OM may therefore represent more than a symptomatic adverse event; it can become a clinically meaningful breach in mucosal defenses at a time when host immunity is markedly compromised, potentially linking local toxicity to systemic complications such as bloodstream infections and febrile neutropenia.^[3]^

Standardized and reliable OM assessment is crucial for both clinical management and research. The National Cancer Institute (NCI) Common Terminology Criteria for Adverse Events (CTCAE) provides widely adopted definitions for OM severity and is frequently used for adverse event reporting in oncology trials and clinical practice.^[4]^ The World Health Organization OM scale is also commonly used because it integrates functional impact (e.g., ability to eat) with clinical findings. However, the availability of multiple grading systems can complicate cross-study comparisons; work examining concordance across OM scales underscores the importance of consistent scale selection and rater training when evaluating OM outcomes and comparing results across settings.^[5]^

International practice guidelines underscore that “basic oral care” is foundational to OM prevention and management across cancer therapies. The Supportive Care in Cancer/International Society of Oral Oncology clinical practice guidelines synthesize extensive evidence and support structured oral assessment, gentle mechanical cleaning (soft toothbrush when tolerated), bland rinses (such as saline and/or sodium bicarbonate), maintenance of oral hydration/moisture, and patient education to reinforce adherence.^[6]^ European Society for Medical Oncology guidelines similarly emphasize supportive oral care and standardized symptom management strategies as key components of mucositis care pathways.^[7]^ Together, these recommendations highlight an important implementation issue: oral care is generally feasible and low cost, yet its potential benefit depends heavily on consistent execution and timely escalation when early mucosal changes are detected.

Despite this consensus, oral care delivery during leukemia induction chemotherapy is often heterogeneous in inpatient practice. Variability may stem from differences in staff training, workload constraints, shift-to-shift practice patterns, and inconsistent documentation and accountability. Patients themselves may have limited capacity to maintain oral hygiene due to fatigue, nausea, mucosal pain, or psychological distress, further reducing adherence. Importantly, the oral cavity is increasingly recognized as a clinically meaningful contributor to infectious risk in neutropenic patients, and mucosal injury may facilitate microbial translocation and systemic infectious complications.^[8]^

Standardizing oral care through a protocol or “bundle” offers a practical strategy to reduce practice variation and improve implementation fidelity. A standardized protocol can define the frequency and method of oral assessment, specify gentle hygiene and rinse practices, integrate moisturizing care, and formalize education and adherence monitoring within routine workflows. Such standardization may be particularly relevant in leukemia induction, where OM risk is high and preventive supportive care is desirable. Nevertheless, the intensity and invasiveness of oral interventions require careful calibration in neutropenic patients. Notably, a randomized trial in acute leukemia reported that an intensive oral hygiene intervention strategy during therapy was not superior in reducing mucositis frequency and was associated with higher local and systemic infection rates, raising concern that overly aggressive or manipulative oral procedures might be detrimental in certain contexts.^[9]^ These findings reinforce the need for pragmatic evaluation of oral care strategies that are standardized yet noninvasive, safe, and feasible for routine inpatient delivery.

The broader evidence base suggests that preventive and supportive interventions can reduce OM, but effects vary by cancer population, treatment intensity, and intervention components. Systematic reviews, including Cochrane evidence syntheses, emphasize that effectiveness is context-dependent and that robust clinical evaluation remains necessary (particularly in high-risk groups such as hematologic malignancies receiving intensive chemotherapy).^[9]^ Additionally, foundational work has highlighted the temporal relationship between mucositis and neutropenia and the potential for mucosal injury to influence infectious outcomes, further supporting the importance of optimized supportive-care pathways during induction chemotherapy.^[10]^

Against this background, we conducted a clinical retrospective study to examine the association between implementation of a standardized oral care protocol and OM severity among patients with leukemia receiving induction chemotherapy. Specifically, we compared OM severity outcomes between patients managed under a standardized oral care protocol and those receiving usual care during the study period. By leveraging routinely collected clinical data, this real-world evaluation aims to inform supportive-care practice and provide evidence on whether standardized, guideline-consistent oral care delivery is associated with improved OM-related outcomes in the leukemia induction setting.

## 2. Methods

### 2.1. Study design and setting

This study was approved by the Ethics Committee of Huanggang Central Hospital. This was a single-center retrospective cohort study conducted in the hematology ward. Consecutive adult patients with leukemia who received induction chemotherapy between September 2024 and September 2025 were identified from electronic medical records.

### 2.2. Participants

Eligible patients were required to meet all of the following inclusion criteria: age ≥ 18 years, diagnosis of leukemia, receipt of inpatient induction chemotherapy during the study period, and availability of nursing oral assessment forms documented during hospitalization. All consecutive patients meeting the eligibility criteria during the study period were included, and no additional sampling or matching procedure was applied.

Patients were excluded if they met either of the following exclusion criteria: baseline OM of CTCAE grade ≥ 2 before initiation of induction chemotherapy, or insufficient documentation to ascertain OM outcomes during hospitalization.

**Figure 1.**
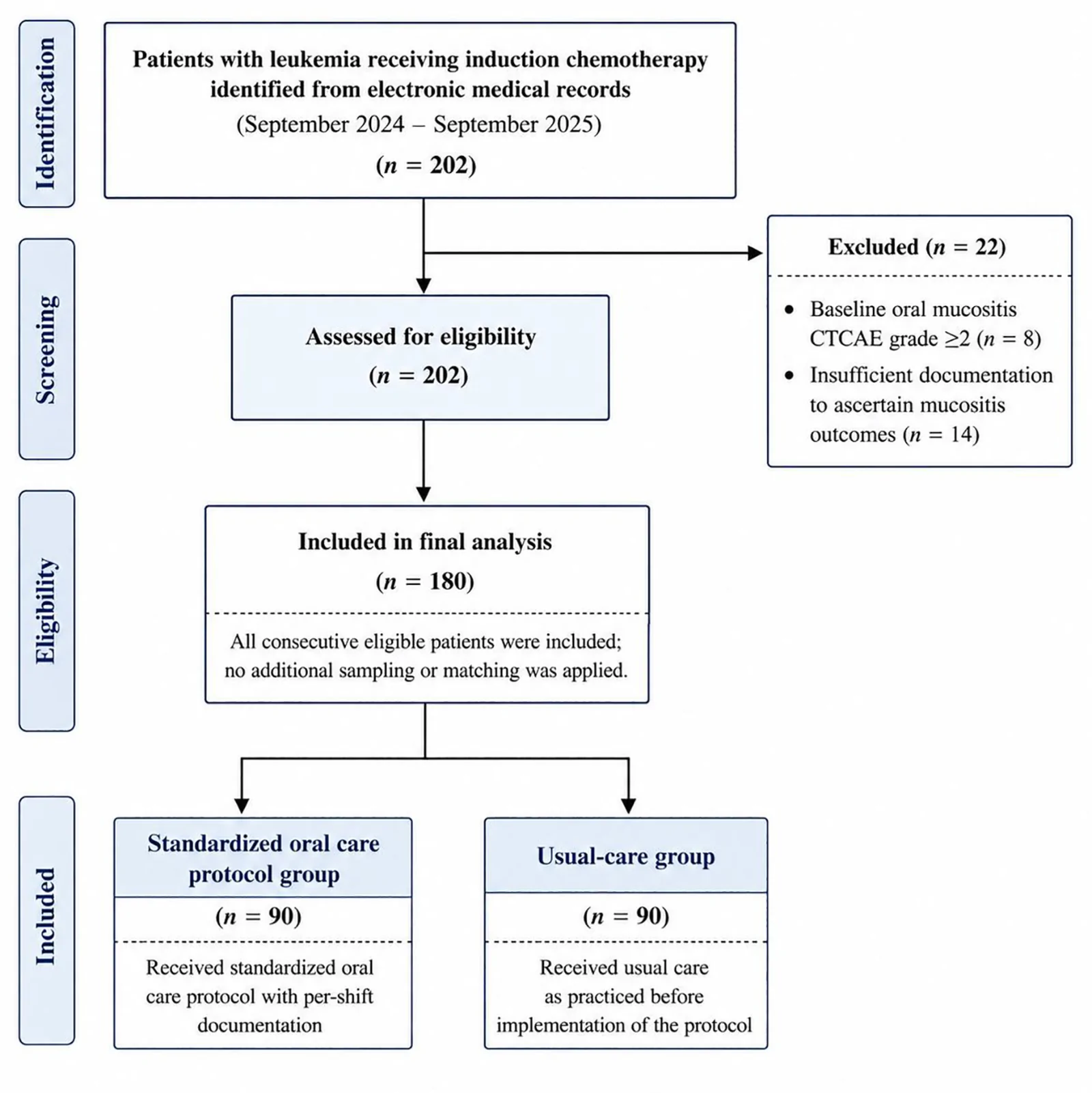
Flow diagram of patient selection. CTCAE = Common Terminology Criteria for Adverse Events, n = number of patients.

A total of 202 patients were screened for eligibility during the study period. Of these, 22 patients were excluded, including 8 patients with baseline OM of CTCAE grade ≥ 2 and 14 patients with insufficient documentation to ascertain mucositis outcomes. The remaining 180 patients were included in the final analysis, with 90 patients in the standardized oral care protocol group and 90 patients in the usual-care group (Fig. 1).

### 2.3. Induction chemotherapy regimen

For patients with acute myeloid leukemia (AML), standard induction chemotherapy consisted of the “7 + 3” regimen, defined as cytarabine 100 to 200 mg/m^2^/day by continuous intravenous infusion on days 1 to 7 plus an anthracycline (daunorubicin 60 mg/m^2^/day [or idarubicin 12 mg/m^2^/day]) on days 1–3, per institutional practice. For patients with acute lymphoblastic leukemia, induction therapy followed an institutional multi-agent protocol (e.g., Hyper-CVAD or pediatric-inspired regimen), and regimens were categorized a priori by intensity (high vs. non-high) for adjusted analyses. All chemotherapy regimen details (agents, doses, and schedule) were abstracted from electronic orders.

### 2.4. Exposure: standardized oral care protocol

The standardized oral care protocol was implemented as a ward-based nursing bundle and applied throughout induction hospitalization (from induction day 1 until discharge). Patients admitted before implementation received usual care, whereas patients admitted on or after implementation received the standardized protocol; the 2 approaches did not coexist routinely during the same calendar period. The protocol consisted of 4 fixed components: structured oral assessment, basic oral hygiene and rinsing, oral moisture care and symptom-triggered escalation, and documentation and adherence monitoring.

#### 2.4.1. Structured oral assessment (each shift)

Nurses performed and documented an oral assessment once per shift using a standardized oral assessment form. Assessment domains included oral pain (0–10 Numeric Rating Scale [NRS]), mucosal erythema/ulceration, bleeding, xerostomia, swallowing difficulty, and ability to tolerate oral intake. OM severity was graded using NCI-CTCAE v5.0 at each assessment.

#### 2.4.2. Basic oral hygiene and rinsing (routine schedule)

Patients received guideline-consistent basic oral care delivered by nurses and/or self-performed with nursing supervision: toothbrushing with a soft toothbrush twice daily (morning and evening); bland mouth rinses (e.g., 0.9% saline and/or sodium bicarbonate solution) 4 times daily and after meals, with instructions to swish gently for 30 to 60 seconds and spit (no vigorous gargling); and lip and oral mucosa care using a water-based moisturizer at least twice daily. Mechanical trauma to the mucosa was minimized; patients were instructed to avoid alcohol-based mouthwashes, spicy/hard foods, and aggressive flossing during neutropenia.

#### 2.4.3. Symptom-triggered escalation and safety rules

Escalation measures were triggered by worsening symptoms or higher OM grades documented on the assessment form. For CTCAE grade ≥ 2 or moderate-to-severe pain (NRS ≥ 4), the protocol required intensified rinsing frequency and physician notification for analgesia optimization and dietary adjustment. For CTCAE grade ≥ 3, the protocol required prompt physician review and supportive measures, including pain-management escalation, nutritional support evaluation, and close monitoring for bleeding or infection. For fever > 38.3°C, nurses initiated the ward febrile-neutropenia pathway and informed the treating team immediately; oral findings were documented as a potential infection source. The protocol explicitly avoided invasive oral procedures during profound cytopenia; no routine debridement or intensive mechanical manipulation was performed.

#### 2.4.4. Documentation and adherence monitoring

Completion of oral care tasks (toothbrushing, rinses, moisturizer) and each-shift assessment were recorded on the protocol checklist. Charge nurses conducted daily checklist verification, and missed care items were addressed during shift handover. Patients not managed under the standardized protocol received usual care at clinician discretion without a mandatory checklist or fixed frequency requirements.

### 2.5. OM assessment

OM severity was graded using NCI-CTCAE v5.0 by ward nurses and recorded on a standardized nursing oral assessment form each shift. The observation window for outcomes was from day 1 of induction chemotherapy to hospital discharge.

### 2.6. Outcomes

The primary outcome was peak OM severity, defined as the highest CTCAE grade recorded during the observation window. Severe OM was defined as CTCAE grade ≥ 3.

Secondary outcomes included: any OM: occurrence of CTCAE grade ≥ 1 during hospitalization; time to onset: days from induction day 1 to the first record of CTCAE grade ≥ 1, calculated among patients who developed OM; duration: number of days from OM onset to resolution (first record of CTCAE grade 0–1 after OM onset), calculated among patients with OM who resolved before discharge; time to resolution: days from induction day 1 to resolution (first record of CTCAE grade 0–1 after OM onset), calculated among patients with OM who resolved before discharge, patients without OM were not included in onset/duration/resolution summaries, and patients who did not resolve before discharge were censored at discharge and excluded from duration/time-to-resolution summaries; symptom burden and supportive care: maximum oral pain score (0–10 NRS) during hospitalization, opioid use (any use, yes/no), cumulative opioid consumption converted to morphine milligram equivalents over hospitalization, and use of parenteral nutrition (yes/no); and infection and utilization: febrile neutropenia (yes/no), defined as a body temperature > 38.3°C with neutropenia consistent with induction chemotherapy, blood culture positivity (yes/no), intensive care unit (ICU) transfer (yes/no), and length of stay (days) calculated from admission to discharge.

### 2.7. Data collection

Data were extracted from electronic medical records, including age, sex, leukemia subtype (AML vs non-AML), induction intensity (high-intensity vs non-high-intensity), baseline OM grade (CTCAE 0–1), smoking history, prophylactic antimicrobial use, laboratory indicators (absolute neutrophil count [ANC] nadir and albumin), nursing-recorded mucositis grades each shift, pain scores, medication administration records for opioids, parenteral nutrition records, microbiology results, ICU transfer, and discharge dates.

### 2.8. Statistical analysis

Baseline characteristics were summarized using median (interquartile range) for non-normally distributed continuous variables, mean ± standard deviation for approximately normally distributed variables, and as counts (%) for categorical variables. Between-group comparisons used the Mann –Whitney *U* test or Student *t* test for continuous variables as appropriate, and the Pearson *χ*^2^ test for categorical variables; Fisher exact test was used when expected cell counts were < 5.

For the primary binary outcome of severe OM (CTCAE grade ≥ 3), univariable odds ratios (ORs) with 95% confidence intervals (CIs) were calculated. A multivariable logistic regression model was subsequently fitted to estimate the independent association between exposure to the standardized oral care protocol and severe OM.

The multivariable model included protocol exposure as the primary independent variable and 5 prespecified clinically relevant covariates: age, leukemia subtype (AML vs non-AML), induction chemotherapy intensity (high vs non-high), baseline OM grade (CTCAE grade 0 vs 1), and serum albumin. These covariates were selected a priori on the basis of their potential clinical association with mucosal vulnerability or chemotherapy-related mucosal injury and their potential to confound the association between oral care protocol exposure and severe OM. Covariate selection was based on clinical relevance rather than univariable statistical significance, and no automated or *P* value–driven stepwise selection procedure was used.

Age was included because mucosal tolerance, tissue repair capacity, and general physiologic reserve may vary with age. Leukemia subtype was included because patients with AML and other leukemia subtypes may differ in disease characteristics, treatment regimens, and supportive-care requirements. Induction chemotherapy intensity was included because the degree of cytotoxic exposure is directly relevant to the risk and severity of treatment-related mucosal injury. Baseline OM grade was included to account for preexisting differences in oral mucosal status at the start of induction chemotherapy. Serum albumin was included as a clinically relevant marker of nutritional and systemic status that may be associated with mucosal integrity and recovery. Sex, smoking history, prophylactic antimicrobial use, and ANC nadir were collected as additional clinical variables but were not included in the primary adjusted model in order to maintain a parsimonious model relative to the number of severe OM events and to reduce the risk of model overfitting. In particular, ANC nadir occurs during the post-induction observation period rather than representing a strictly baseline characteristic and was, therefore, not selected as a primary baseline adjustment variable.

No formal adjustment for multiple comparisons was applied to secondary outcomes. These secondary analyses were considered exploratory and should, therefore, be interpreted cautiously, particularly when *P* values were close to the conventional significance threshold. Statistical significance for the primary analysis was defined as a 2-sided *P* < .05. Analyses were performed using Statistical Package for the Social Sciences Statistics for Windows, version 27.0 (International Business Machines Corp.).

## 3. Results

### 3.1. Patient characteristics (Table 1)

A total of 180 patients were included, with 90 in the standardized oral care protocol group and 90 in the usual-care group. Baseline characteristics were generally comparable between groups, including age, sex, leukemia subtype, induction intensity, baseline CTCAE OM grade (0–1), prophylactic antimicrobials, and key laboratory indicators (Table 1).

**Table 1:** Baseline characteristics.

Characteristic	Protocol (n = 90)	Usual care (n = 90)	*P* value
Age, yrs, median (IQR)	50 (38–61)	48 (36–60)	.53
Male, n (%)	50 (55.6)	52 (57.8)	.76
AML, n (%)	68 (75.6)	66 (73.3)	.72
High-intensity induction regimen, n (%)	61 (67.8)	59 (65.6)	.75
Baseline oral mucositis CTCAE grade 0, n (%)	79 (87.8)	80 (88.9)	.82
Baseline oral mucositis CTCAE grade 1, n (%)	11 (12.2)	10 (11.1)	.82
Smoking history (current/former), n (%)	18 (20.0)	20 (22.2)	.72
Prophylactic antimicrobials, n (%)	64 (71.1)	62 (68.9)	.74
ANC nadir (× 10^9^/L), median (IQR)	0.04 (0.02–0.07)	0.05 (0.02–0.08)	.41
Albumin, g/L, mean ± SD	36.8 ± 4.5	36.2 ± 4.7	.39

AML = acute myeloid leukemia, ANC = absolute neutrophil count, CTCAE = Common Terminology Criteria for Adverse Events, IQR = interquartile range, n = number of patients, SD = standard deviation.

### 3.2. Primary outcome: peak OM severity (Table 2)

Peak CTCAE OM severity during hospitalization differed between groups (Table 2). Severe OM (CTCAE grade ≥ 3) occurred in 17/90 (18.9%) patients in the protocol group versus 30/90 (33.3%) in the usual-care group (OR 0.46, 95% CI 0.24–0.89, *P* = .027). In multivariable analysis, protocol receipt remained associated with lower odds of severe mucositis (adjusted OR 0.50, 95% CI 0.25–0.98, *P* = .044). The overall distribution of peak CTCAE grades did not differ significantly between groups (*P* = .197, Pearson *χ*^2^ test).

**Table 2:** Primary outcome and peak CTCAE grade distribution.

Outcome	Protocol (n = 90)	Usual care (n = 90)	Effect estimate	*P* value
Severe OM (CTCAE ≥ 3), n (%)	17 (18.9)	30 (33.3)	OR 0.46 (0.24–0.89)	.027
Adjusted severe OM (multivariable)	-	-	aOR 0.50 (0.25–0.98)	.044
Peak CTCAE grade distribution, n (%)			-	.197
Grade 0	9 (10.0)	6 (6.7)		
Grade 1	27 (30.0)	18 (20.0)		
Grade 2	37 (41.1)	36 (40.0)		
Grade 3	15 (16.7)	26 (28.9)		
Grade 4	2 (2.2)	4 (4.4)		

aOR = adjusted odds ratio, CTCAE = Common Terminology Criteria for Adverse Events, n = number of patients, OM = oral mucositis, OR = odds ratio.

### 3.3. Time-course outcomes: incidence and duration (Table 3)

OM of any grade (CTCAE ≥ 1) occurred in 74/90 (82.2%) patients in the protocol group and 80/90 (88.9%) in usual care (*P* = .20). Median time to OM onset was 7 (5–10) days in the protocol group versus 6 (4–9) days in usual care (*P* = .18). Median OM duration was shorter in the protocol group (6 [3–9] vs 8 [5–12] days, *P* = .003). Time to resolution was also shorter (14 [11–18] vs 16 [12–20] days, *P* = .04) (Table 3).

**Table 3:** Incidence and duration.

Outcome (until discharge)	Protocol (n = 90)	Usual care (n = 90)	*P* value
OM any grade (CTCAE ≥ 1), n (%)	74 (82.2)	80 (88.9)	.20
Time to OM onset, d, median (IQR)	7 (5–10)	6 (4–9)	.18
OM duration, d, median (IQR)	6 (3–9)	8 (5–12)	.003
Time to resolution, d, median (IQR)	14 (11–18)	16 (12–20)	.04

CTCAE = Common Terminology Criteria for Adverse Events, IQR = interquartile range, n = number of patients, OM = oral mucositis.

### 3.4. Symptom burden and supportive-care (Table 4)

Maximum oral pain scores were lower in the protocol group (median 3 [2–5]) compared with usual care (median 5 [3–6], *P* < .001). Fewer patients required opioids in the protocol group (40/90 [44.4%] vs 54/90 [60.0%], *P* = .036), and cumulative opioid consumption (morphine milligram equivalents) was lower (60 [0–150] vs 120 [30–240] mg, *P* = .006). Parenteral nutrition was less frequently used in the protocol group (12/90 [13.3%] vs 22/90 [24.4%], *P* = .049) (Table 4).

**Table 4:** Symptom burden and supportive care.

Outcome	Protocol (n = 90)	Usual care (n = 90)	*P* value
Max daily oral pain (NRS), median (IQR)	3 (2–5)	5 (3–6)	< .001
Any opioid use, n (%)	40 (44.4)	54 (60.0)	.036
Opioid use (MME), mg, median (IQR)	60 (0–150)	120 (30–240)	.006
Parenteral nutrition use, n (%)	12 (13.3)	22 (24.4)	.049

IQR = interquartile range, MME = morphine milligram equivalents, n = number of patients, NRS = Numeric Rating Scale.

### 3.5. Infection and hospital utilization (Table 5)

Febrile neutropenia occurred in 52/90 (57.8%) of the protocol group and 58/90 (64.4%) of the usual-care group (*P* = .36). Blood culture positivity was 10.0% versus 13.3% (*P* = .49). Median length of stay was 24 (20–30) days in the protocol group and 26 (21–33) days in usual care (*P* = .09). ICU transfer was uncommon (3.3% vs 5.6%, *P* = .47) (Table 5).

**Table 5:** Infection and hospital utilization.

Outcome	Protocol (n = 90)	Usual care (n = 90)	*P* value
Febrile neutropenia, n (%)	52 (57.8)	58 (64.4)	.36
Blood culture positive, n (%)	9 (10.0)	12 (13.3)	.49
Length of stay, d, median (IQR)	24 (20–30)	26 (21–33)	.09
ICU transfer, n (%)	3 (3.3)	5 (5.6)	.47

ICU = intensive care unit, IQR = interquartile range, n = number of patients.

## 4. Discussion

In this retrospective cohort study of hospitalized patients undergoing leukemia induction chemotherapy, receipt of a standardized oral care protocol was associated with a pattern of improved OM outcomes assessed using NCI-CTCAE v5.0 grading documented on nursing oral assessment forms each shift.^[11]^ Compared with usual care, the protocol group demonstrated findings consistent with reduced mucositis burden, including a lower likelihood of severe OM (CTCAE grade ≥ 3) and a shift toward lower peak mucositis grades. Secondary analyses suggested improvement in clinically meaningful domains such as mucositis course (shorter duration and/or faster resolution), symptom burden (lower maximum oral pain scores), and downstream supportive-care needs (reduced opioid exposure and less frequent parenteral nutrition use). Collectively, these findings support the premise that standardizing core oral care practices during the high-risk induction period may translate into measurable clinical benefits.

The observed direction of effect is biologically plausible and aligns with contemporary supportive-care principles. OM is understood as a multistage injury process involving epithelial damage and inflammatory amplification that culminates in barrier disruption and ulceration.^[12]^ In leukemia induction, profound cytopenias may impair mucosal repair and predispose to secondary infection and bleeding, thereby magnifying the clinical impact of mucosal breakdown.^[13]^ Standardized protocols may be effective through 2 complementary mechanisms. First, structured, high-frequency oral assessment (each shift in our setting) increases the likelihood of early detection of erythema, edema, or focal breakdown, enabling timely escalation of supportive measures before lesions progress. Second, consistent delivery of foundational oral care (gentle mechanical cleansing when tolerated, bland rinses, moisturization, and patient education) can reduce local irritants, maintain mucosal hydration, and support barrier integrity. These components are consistent with international guideline frameworks that emphasize basic oral care and systematic assessment as the cornerstone of mucositis prevention and management.^[14]^

Our results also underscore the importance of implementation fidelity in routine inpatient practice. Although many institutions endorse “basic oral care,” real-world delivery during busy ward care can be heterogeneous, with variability across staff, shifts, and documentation practices. Education and workflow-focused interventions aimed at improving nurses’ knowledge and skills in oral care have been evaluated in hematology settings, highlighting that competence and consistency are prerequisites for reliable oral care delivery.^[15]^ Thus, the protocol can be viewed as both a clinical intervention and an implementation strategy: it reduces unwarranted practice variation by defining assessment frequency and content, specifying recommended interventions, and embedding education and adherence documentation into routine nursing care.

Potential improvements in pain and supportive-care utilization are clinically meaningful. Oral pain is a major driver of reduced oral intake, sleep disruption, diminished quality of life, and increased analgesic requirements. In hematologic malignancies, mucositis has been associated with a higher need for opioid analgesia and nutritional support.^[16]^ Accordingly, an intervention that attenuates mucositis severity may decrease symptom burden and reduce the intensity of supportive care. At the same time, broader outcomes such as febrile neutropenia and length of stay are multifactorial in induction chemotherapy and may be less sensitive to changes in mucositis alone.

Safety considerations are particularly salient in profoundly immunosuppressed and thrombocytopenic patients. Oral care practices that are overly aggressive or traumatic may theoretically worsen mucosal injury or introduce infection risk. The standardized protocol evaluated here emphasized gentle, nontraumatic care rather than intensive mechanical manipulation, which may help balance feasibility and safety in routine implementation. Evidence from adults with acute leukemia receiving induction chemotherapy has compared commonly used mouth care agents such as sodium bicarbonate solution and chlorhexidine mouthwash, reinforcing the need for feasible, low-irritant strategies tailored to tolerance.^[17]^ More broadly, systematic reviews conclude that evidence supporting many prophylactic agents is variable and context-specific, supporting a pragmatic focus on safe foundational care while selecting adjuncts based on local evidence and patient acceptability.^[18]^

This study has several strengths. It reflects real-world inpatient care during leukemia induction, a period characterized by a high mucositis risk and a substantial patient burden. Mucositis grading was captured using standardized nursing assessment forms completed each shift, providing relatively high temporal granularity for evaluating onset, peak severity, and resolution. Standardized oral assessment instruments have long been advocated in oncology care; for example, the oral assessment guide supports structured evaluation of oral cavity changes over time,^[19]^ and subsequent work has emphasized the importance of reliable measurement for both clinical management and research.^[20]^ In addition, we prespecified clinically relevant covariates and used adjusted analyses to address confounding, which is essential in observational comparisons.

Nevertheless, limitations inherent to the retrospective design must be considered. Residual confounding may persist despite adjustment, including unmeasured baseline dental status, oral microbiome differences, nutritional reserve, variations in analgesic prescribing practices, and concurrent supportive interventions. Misclassification is possible if protocol exposure or adherence was incompletely documented. Although CTCAE-based grading provides a standardized framework,^[21]^ inter-rater variability across nurses and shifts may influence recorded severity. Pain scores and opioid exposure are clinically meaningful but can be influenced by institutional pain-management culture and patient preferences. The single-center setting and local workflow characteristics may also limit generalizability. Finally, multiple secondary outcomes were exploratory, no multiplicity adjustment was performed, and borderline *P* values should therefore be interpreted cautiously.

These findings have practical implications for clinical care and quality improvement. A standardized oral care pathway can be implemented with modest resources by leveraging nursing workflows and documentation tools already present in many hematology wards. Implementation should include staff training on CTCAE grading,^[22]^ clear criteria for escalation (when to involve dentistry/oral medicine), and monitoring of adherence. Future work could adopt prospective pragmatic designs to confirm effectiveness and identify the most impactful protocol components. Multicenter studies would enhance external validity and enable subgroup analyses by leukemia subtype, induction intensity, baseline oral risk, and cytopenia depth. Incorporating patient-reported outcomes and quality-of-life measures would further clarify the patient-centered value of protocolized oral care, in line with guideline calls for evidence-based supportive-care pathways.

## 5. Conclusion

In this hospital-based study targeting leukemia patients who have undergone induction chemotherapy, a standardized oral care protocol documented each shift was associated with a pattern of improved OM outcomes based on CTCAE v5.0 nursing assessments, including reduced severe mucositis and lower symptom burden. While causal inference is limited by the retrospective observational design, these findings support standardized basic oral care as a feasible, low-cost approach aligned with international mucositis guidance. Prospective multicenter validation and pragmatic implementation studies are warranted to confirm benefit, optimize protocol components, and evaluate downstream effects on supportive-care utilization and patient-reported outcomes.

## Author contributions

**Conceptualization:** Fen Yu, Tian Mao.

**Data curation:** Fen Yu, Tian Mao.

**Formal analysis:** Fen Yu, Tian Mao.

**Funding acquisition:** Tian Mao.

**Investigation:** Tian Mao.

**Writing** – **original draft:** Tian Mao.

**Writing** – **review & editing:** Tian Mao.
